# SbMYB3 transcription factor promotes root-specific flavone biosynthesis in *Scutellaria baicalensis*

**DOI:** 10.1093/hr/uhac266

**Published:** 2022-12-02

**Authors:** Yumin Fang, Jie Liu, Minmin Zheng, Sanming Zhu, Tianlin Pei, Mengying Cui, Lijing Chang, Hanwen Xiao, Jun Yang, Cathie Martin, Qing Zhao

**Affiliations:** Shanghai Key Laboratory of Plant Functional Genomics and Resources, Shanghai Chenshan Botanical Garden, Shanghai, 201602, China; Shanghai Key Laboratory of Plant Functional Genomics and Resources, Shanghai Chenshan Botanical Garden, Shanghai, 201602, China; Shanghai Key Laboratory of Plant Functional Genomics and Resources, Shanghai Chenshan Botanical Garden, Shanghai, 201602, China; State Key Laboratory of Plant Molecular Genetics, CAS Center for Excellence in Molecular Plant Sciences, Chinese Academy of Sciences, Shanghai, 200032, China; National Key Laboratory of Crop Biology, College of Life Sciences, Shandong Agricultural University, Taian, 271000, China; Shanghai Key Laboratory of Plant Functional Genomics and Resources, Shanghai Chenshan Botanical Garden, Shanghai, 201602, China; State Key Laboratory of Plant Molecular Genetics, CAS Center for Excellence in Molecular Plant Sciences, Chinese Academy of Sciences, Shanghai, 200032, China; Shanghai Key Laboratory of Plant Functional Genomics and Resources, Shanghai Chenshan Botanical Garden, Shanghai, 201602, China; State Key Laboratory of Plant Molecular Genetics, CAS Center for Excellence in Molecular Plant Sciences, Chinese Academy of Sciences, Shanghai, 200032, China; Shanghai Key Laboratory of Plant Functional Genomics and Resources, Shanghai Chenshan Botanical Garden, Shanghai, 201602, China; Shanghai Key Laboratory of Plant Functional Genomics and Resources, Shanghai Chenshan Botanical Garden, Shanghai, 201602, China; State Key Laboratory of Plant Molecular Genetics, CAS Center for Excellence in Molecular Plant Sciences, Chinese Academy of Sciences, Shanghai, 200032, China; John Innes Centre, Norwich NR4 7UH, UK; Shanghai Key Laboratory of Plant Functional Genomics and Resources, Shanghai Chenshan Botanical Garden, Shanghai, 201602, China; State Key Laboratory of Plant Molecular Genetics, CAS Center for Excellence in Molecular Plant Sciences, Chinese Academy of Sciences, Shanghai, 200032, China

## Abstract

*Scutellaria baicalensis* Georgi produces abundant root-specific flavones (RSFs), which provide various benefits to human health. We have elucidated the complete biosynthetic pathways of baicalein and wogonin. However, the transcriptional regulation of flavone biosynthesis in *S. baicalensis* remains unclear. We show that the SbMYB3 transcription factor functions as a transcriptional activator involved in the biosynthesis of RSFs in *S. baicalensis*. Yeast one-hybrid and transcriptional activation assays showed that SbMYB3 binds to the promoter of *flavone synthase II-2* (*SbFNSII-2*) and enhances its transcription. In *S. baicalensis* hairy roots, RNAi of *SbMYB3* reduced the accumulation of baicalin and wogonoside, and *SbMYB3* knockout decreased the biosynthesis of baicalein, baicalin, wogonin, and wogonoside, whereas *SbMYB3* overexpression enhanced the contents of baicalein, baicalin, wogonin, and wogonoside. Transcript profiling by qRT–PCR demonstrated that SbMYB3 activates *SbFNSII-2* expression directly, thus leading to more abundant accumulation of RSFs. This study provides a potential target for metabolic engineering of RSFs.

## Introduction

Medicinal plants produce various specialized metabolites, some of them having remarkable activities against diseases, such as artemisinin [1], tanshinones [2], and baicalein [3, 4]. *Scutellaria baicalensis* Georgi, also known as Huang-Qin, has been used for medicine for more than 2000 years [5]. Its major bioactive components are root-specific flavones (RSFs), which have pharmacological activities in liver protection, anti-oxidation, anti-inflammation, anti-respiratory syncytial virus, anti-mutagenesis, anti-cancer, neuroprotection, and anti-anxiety. Baicalein, baicalin, wogonin, and wogonoside are the main RSFs with anti-tumor activity [6, 7]. Among them, baicalein is a strong inhibitor of the 3C-like protease of SARS-CoV-2 [3, 4].

Both the aerial parts and the roots of *S. baicalensis* produce flavones. The flavones of aerial organs comprise mainly scutellarein and scutellarin, whereas the most abundant flavones of the roots are baicalein, baicalin, wogonin, and wogonoside (RSFs) [5, 8]. We have elucidated the biosynthetic pathways of flavones in *S. baicalensis*. There are two flavone biosynthetic pathways in *S. baicalensis*, including the classic flavone biosynthetic pathway operating in aerial organs and the RSF pathway operating in the roots. The RSF pathway likely evolved from the classical pathway [9, 10]. The classical flavone biosynthesis pathway uses phenylalanine produced by the shikimate pathway [11]. Phenylalanine is sequentially catalyzed by five enzymes to form naringenin: phenylalanine ammonia lyase (SbPAL), cinnamate 4-hydroxylase (SbC4H), 4-coumaroyl:CoA-ligase (SbCLL-1), chalcone synthase (SbCHS-1), and chalcone isomerase (SbCHI). Then, flavone synthase II-1 (SbFNSII-1) converts naringenin to apigenin, which is then hydroxylated by flavone 6- hydroxylase (SbF6H) to form scutellarein [5, 8, 10]. The biosynthetic pathway of RSFs also uses phenylalanine to form pinocembrin, requiring the catalytic activity of four enzymes: SbPAL, a specific cinnamate CoA ligase (SbCLL-7), pinocembrin-chalcone synthase (SbCHS-2), and SbCHI. Pinocembrin is converted by a specific flavone synthase isoform, FNSII-2 (SbFNSII-2), to form chrysin. SbF6H and flavone 8-hydroxylase (SbF8H) hydroxylate chrysin in bifurcating pathways to produce baicalein and norwogonin, respectively [5, 10]. Norwogonin is methylated to form wogonin by an 8-*O*-methyltransferase (SbPFOMT5) [9]. Flavonoid 7-*O*-glucosyltransferase (SbUBGAT) uses UDP-glucuronic acid as the sugar donor and catalyzes baicalein to baicalin [12, 13] (Supplementary Data Fig. S1).

Transcription factors are involved in plant growth, development, and specialized metabolism. Those controlling specialized metabolism usually function by activating or repressing transcription of ‘structural’ genes encoding enzymes of the regulated pathways. The R2R3-MYB family is one of the largest transcription factor families in plants, and plays important roles in plant growth and development, cell morphogenesis, primary metabolism, specialized metabolism, hormone signal transduction, and responses to environmental stresses [14–17]. MYB transcription factors comprise a DNA-binding domain of one to four MYB repeats of 50–53 amino acids, which each form helix, helix turn helix conformations. The R2R3-MYBs are the largest subfamily of MYB transcription factors in plants [11, 17–20]. The function of MYB transcription factors in crops has been studied extensively, but there are only a few related studies in medicinal plants [21]. Some studies have shown that MYB transcription factors from *S. baicalensis* are involved in the regulation of flavonoid synthesis and stress responses in tobacco. Overexpression of *SbMYB2* or *SbMYB7* enhanced phenylpropanoid production and the tolerance of oxidative stress in transgenic tobacco, but decreased the accumulation of dicaffeoylspermidine and quercetin-3,7-*O*-diglucoside [22, 23]. *SbMYB8* regulated flavonoid synthesis and improved plant tolerance to drought in transgenic tobacco, and electrophoretic mobility shift assays showed that this transcription factor could bind to the promoter sequence of *SbCHS-2* [24]. However, the function of these SbMYBs has been studied only in heterologous hosts, and their regulatory functions as transcription factors are still largely unknown in *S. baicalensis*.

We aimed to identify transcription factors involved in regulation of RSF biosynthesis in *S. baicalensis*. We cloned the coding regions of transcription factors highly expressed in roots. Candidate *SbMYB* genes were screened using yeast single hybridization with the RSF-specific *SbFNSII-2* promoter. Our study demonstrated that SbMYB3 can bind to the *SbFNSII-2* promoter and enhances its activity, showing that it is a positive regulator of RSF biosynthesis in roots of *S. baicalensis*.

## Results

### Characterization of *SbMYBs* that are highly expressed in roots

**Figure 1:**
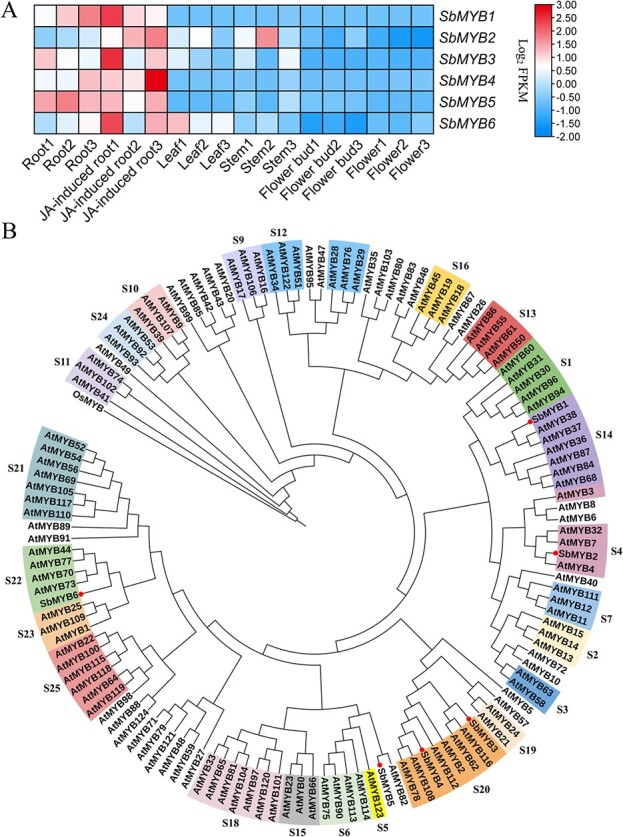
Identification of *SbMYBs* highly expressed in roots. (A) Expression patterns of candidate *SbMYBs*. Expression levels are shown as exponential values with base 2 (fragments per kilobase of transcript per million mapped reads, FPKM), and the scale is shown at the right. All data are shown with three biological replicates. (B) Phylogenetic analysis of SbMYBs and *A. thaliana* MYBs from the different subgroups. Neighbor-joining was used to construct this tree with 1000 replicate bootstrap support. The tree was rooted with an *Oryza sativa* MYB (CAA72218.1). MYB proteins from *S. baicalensis* are marked with a red dot.

*S. baicalensis* accumulates abundant RSFs in its roots, which can be enhanced by jasmonic acid (JA) treatment. Previous studies indicated that JA promoted accumulations of baicalein, baicalin, and wogonin notably in cell suspensions or hairy roots of *S. baicalensis* [5, 8]. To identify transcription factors that potentially regulate the synthesis of RSFs, we analyzed the transcriptomes of different organs of *S. baicalensis* [5]. Genes encoding six MYB proteins were found that were highly expressed in roots and could be induced by JA (Fig. 1A). We isolated full-length cDNAs of the genes using primer pairs based on the loci identified and six *SbMYBs* were obtained, which contain 498- to 936-bp open reading frames (ORFs) encoding proteins of 165–311 amino acids (Supplementary Data Tables S1–S3). Among them, *SbMYB3* contains a 795-bp ORF that encodes a protein of 264 amino acids with predicted molecular weight of 30.4 kDa (Supplementary Data Table S3).

Bioinformatic analysis of the six SbMYBs was carried out to identify the subgroups of R2R3-MYB transcription factors to which they belong. First, we constructed a phylogenetic tree using protein sequences of *Arabidopsis thaliana* MYBs and SbMYBs. Phylogenetic analysis indicated that SbMYB1, SbMYB2, SbMYB3, SbMYB4, and SbMYB6 belong to subgroup 14, 4, 20, 20, and 22 [18], respectively. SbMYB5 is most similar to AtMYB82 [25]. SbMYB3 is most similar to AtMYB62 [26], and shares 50.2% and 44.8% identity with AtMYB62 and AtMYB116 at the amino acid level, respectively (Fig. 1B). Full-length protein sequence alignment of these root-specific SbMYBs and several AtMYBs from different subgroups showed that all these SbMYBs have intact R2 and R3 domains (Supplementary Data Fig. S2) and belong to the R2R3-MYB family.

The biosynthetic pathways of baicalein and wogonin have been elucidated completely, and a gene encoding a specific isoform of flavone synthase II (SbFNSII-2) plays a vital role in their synthesis [5, 9, 10]. To test whether the MYB transcription factors could bind to the *SbFNSII-2* promoter, we also cloned the promoter region of −1985 to +1 upstream of the *SbFNSII-2* coding region. Then, we analyzed the *SbFNSII-2* promoter using the PlantCARE database [27], and found that it contains MYB-binding motifs: MYB-recognition element (MRE, AACCTAA) [28], MYB motif (CAACCA), and MYB binding *cis*-element (MBS, CAACTG) [29]. Moreover, other MYB-binding motifs (CACCCACCG, CACCAAA, and CACCAAA) [30] were also found in the *SbFNSII-2* promoter (Supplementary Data Fig. S3). The results imply that the *SbFNSII-2* promoter is a potential target of an R2R3-MYB transcription factor. We also found a Class II transposable element lying in the promoter of *SbFNSII-2* (−960 to −400 upstream of the *SbFNSII-2* coding region) using the Plant Repeat Database [31], and this type of transposon is a member of the hAT family. Moreover, the MYB motif lies in this transposable element (Supplementary Data Fig. S3), suggesting that this transposon may confer new root-specific regulation due to the MYB-binding site it carries.

### SbMYB3 transcription factor binds to the promoter of *SbFNSII-2*

**Figure 2:**
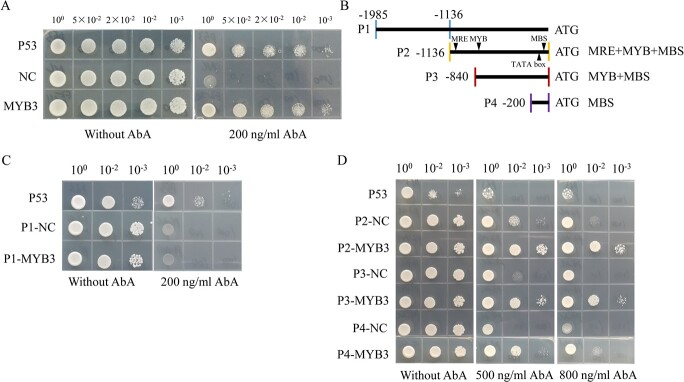
Yeast one-hybrid assays illustrating the interaction between SbMYB3 transcription factor and *SbFNSII-2* promoter. (A) Interaction analysis between SbMYB3 transcription factor and *SbFNSII-2* promoter. (B) Schematic diagram of truncated *SbFNSII-2* promoters. (C) Interaction analysis between SbMYB3 transcription factor and the P1 region of the *SbFNSII-2* promoter. (D) Interaction analysis of SbMYB3 transcription factor and the P2, P3, and P4 regions of *SbFNSII-2* promoter. NC, P1-NC, P2-NC, P3-NC, P4-NC, and P53 represent empty pGADT7 plus the *SbFNSII-2* promoter bait, empty pGADT7 plus the bait of P1 region of *SbFNSII-2* promoter, empty pGADT7 plus the bait of P2 region of *SbFNSII-2* promoter, empty pGADT7 plus the bait of P3 region of *SbFNSII-2* promoter, empty pGADT7 plus the bait of P4 region of *SbFNSII-2* promoter, and the positive control, respectively. AbA, aureobasidin A.

The yeast one-hybrid system was employed to test the potential interaction between the candidate MYBs and native *SbFNSII-2* promoter. The results indicated that only the SbMYB3 transcription factor could bind to the *SbFNSII-2* promoter (Fig. 2A and Supplementary Data Fig. S4). A deletion analysis of the *SbFNSII-2* promoter was carried out to determine the region where SbMYB3 binds on the *SbFNSII-2* promoter. To test whether the MYB-binding motifs are responsible for the interaction between SbMYB3 and *SbFNSII-2* promoter, we selected four regions of the *SbFNSII-2* promoter to construct yeast bait strains: the P1 region (−1985 to −1136) without MYB-binding motifs; the P2 region (−1136 to 0) with MRE, MYB, and MBS motifs; the P3 region (−840 to 0) with MYB and MBS motifs; and the P4 region (−200 to 0) with the MBS motif (Fig. 2B). The results showed that SbMYB3 can bind to regions of P2, P3, and P4 of the *SbFNSII-2* promoter, but cannot bind to the P1 region. In addition, with the decrease in the number of MYB-binding motifs in the promoter region, the strength of interaction was weakened (Fig. 2C and D). These results suggested that the P4 region of the *SbFNSII-2* promoter is adequate for the interaction with the SbMYB3 transcription factor, but the number of MYB-binding motifs determines the strength of this interaction.

In *S. baicalensis*, two genes, *SbFNSII-1.1* and *SbFNSII-1.2*, encode identical proteins (SbFNSII-1) that are responsible for the synthesis of apigenin in the aerial parts of the plant [5]. The *SbFNSII-2* gene lies adjacent to *SbFNSII-1.2* on the same chromosome in the *S. baicalensis* genome, in a tail-to-tail inverted duplication, suggesting that *SbFNSII-2* was produced by duplication of *SbFNSII-1.2* [9]. The presence of the Class II DNA transposon in the promoter of *SbFNSII-2* suggests that the duplication may have arisen as a result of aberrant transposition of a hAT transposon, Tam3, as described at the *nivea* locus in *Antirrhinum majus* [32]. We checked whether SbMYB3 could bind to the *SbFNSII-1.2* promoter from −1995 to +1 upstream of the ATG start codon of *SbFNSII-1.2* using yeast one-hybrid assays (Supplementary Data Figs S5 and S6). Although the *SbFNSII-1.2* promoter contains several typical MYB-binding motifs, the SbMYB3 transcription factor could not bind to the *SbFNSII-1.2* promoter, although it bound to the *SbFNSII-2* promoter (Supplementary Data Figs S4–S6). These data revealed that SbMYB3 may be a transcription factor that evolved to regulate the RSF pathway. No Class II transposable element was found in the promoter region of *SbFNSII-1.2*.

**Figure 3:**
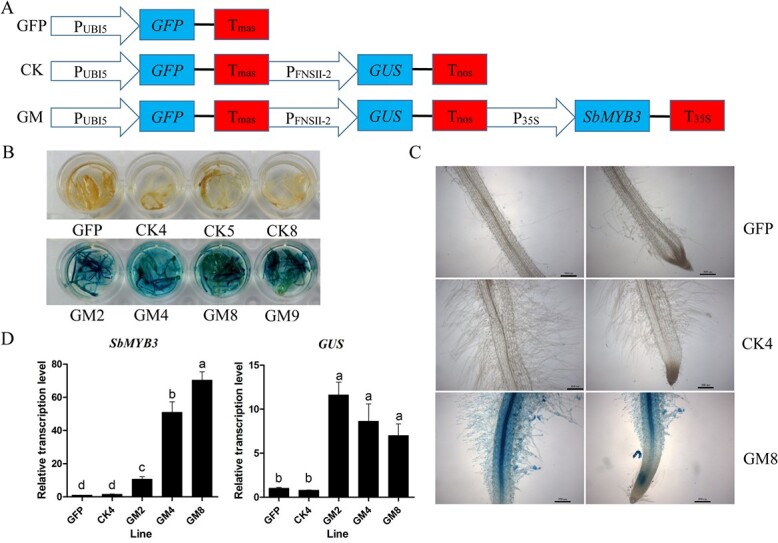
GUS histochemical assays of *N. tabacum* hairy roots demonstrating that SbMYB3 transcription factor *trans*-activates the *SbFNSII-2* promoter. (A) Diagram of plant expression vectors. GFP, CK, and GM represent the vector only expressing GFP protein; the vector expressing GFP and GUS driven by the *SbFNSII-2* promoter; and the vector expressing GFP and GUS driven by the *SbFNSII-2* promoter and SbMYB3, respectively. (B) and (C) GUS histochemical assays of GFP, CK, and GM lines. (D) Relative expression levels of *SbMYB3* and *GUS* in the different groups. All data are the means of three biological replicates; error bars indicate the standard deviation. Significance was determined using the least significance difference (LSD), and different letters above the bars indicate significantly different values (*P* < .05).

To verify whether SbMYB3 is a transcriptional activator in plants, β-galactosidase (GUS) histochemical assays of *Nicotiana tabacum* hairy roots were performed. We designed three vectors: the first vector expressed only green fluorescent protein (GFP) driven by the UBI5 promoter (GFP group); the second vector expressed GFP driven by the UBI5 promoter and GUS driven by the *SbFNSII-2* promoter (CK group); and the last vector expressed the same DNA regions as the second vector plus SbMYB3 driven by the CaMV35S promoter (GM group). The first and second vectors were chosen as the control groups (Fig. 3A). Then, hairy roots were induced from tobacco leaf explants transformed with *Agrobacterium rhizogenes* Ar1193 containing the vectors. After treatment with GUS staining solution, *N. tabacum* hairy roots of the GM group were stained dark blue, whereas hairy roots of the GFP group and CK groups showed no color change (Fig. 3B and C). This work showed that SbMYB3 could *trans*-activate the *SbFNSII-2* promoter, thus driving *GUS* expression. The expression patterns of *SbMYB3* and *GUS* were consistent with the coloration results (Fig. 3B–D). The expression of *GUS* was dramatically upregulated in the GM lines, and was 7- to 12-fold higher than that in the control lines (Fig. 3D). These data suggested that the SbMYB3 transcription factor can activate the *SbFNSII-2* promoter, supporting its regulatory role in RSF biosynthesis.

### SbMYB3 is localized in the nucleus

To determine the subcellular localization of SbMYB3, the coding region of *SbMYB3* was fused in-frame to the N terminus of GFP, and the resulting construct, *35Spro*::*SbMYB3*-*GFP* along the with *35Spro*::*GFP* empty vector (EV) were transiently expressed in tobacco leaves. The results indicated that the fluorescent signal of the SbMYB3-GFP fusion protein was predominantly observed in the nucleus, and green fluorescence emitted from the SbMYB3-GFP fusion protein matched the blue fluorescence produced by DAPI (4′,6-diamidino-2-phenylindole) staining of nuclei, whereas the signal of 35S-GFP was detected in the nucleus and cytoplasm (Supplementary Data Fig. S7), suggesting that SbMYB3 is a nucleus-localized protein.

### RNAi and knockout of *SbMYB3* reduced RSF biosynthesis in *S. baicalensis* hairy roots

**Figure 4:**
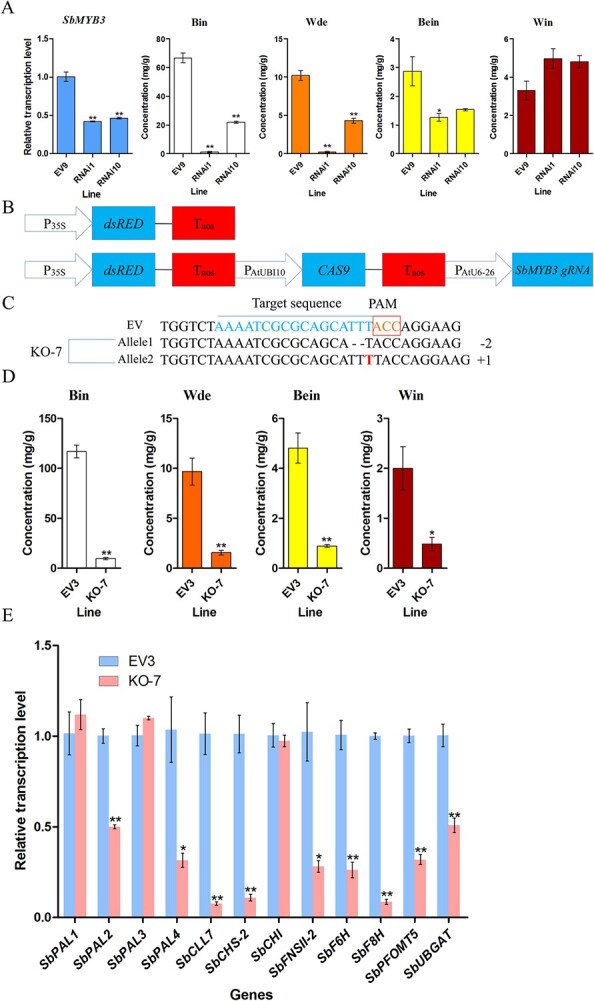
RNAi and knockout of *SbMYB3* decreased accumulations of root-specific flavones in *S. baicalensis* hairy roots. (A) Expression analysis of *SbMYB3* and contents of baicalin, wogonoside, baicalein, and wogonin in *S. baicalensis* hairy roots. EV9 and RNAi represent hairy roots of the control line and *SbMYB3*-RNAi lines, respectively. Bin, Bein, Wde, and Win represent baicalin, baicalein, wogonoside, and wogonin, respectively. (B) Diagram of plant expression vectors. (C) Gene editing results of the target sequence of *SbMYB3*. (D) Contents of baicalin, baicalein, wogonoside, and wogonin in hairy roots of the control line and knockout line. KO-7 and EV3 represent hairy roots of the *SbMYB3*-knockout line and the control line, respectively. (E) Relative expression levels of key genes involved in root-specific flavone biosynthesis in *S. baicalensis* hairy roots. *SbPAL1*, *SbPAL2*, *SbPAL3*, and *SbPAL4* encode phenylalanine ammonia lyases. *SbCLL7*, *SbCHS-2*, *SbCHI*, *SbFNSII-2*, *SbF6H*, *SbF8H*, *SbPFOMT5*, and *SbUBGAT* encode 4-coumaroyl:CoA-ligase, pinocembrin-chalcone synthase, chalcone isomerase, flavone synthase II-2, flavone 6- hydroxylase, flavone 8-hydroxylase, 8-*O*-methyl transferase, and flavonoid 7-*O*-glucosyltransferase, respectively. All data are means of three biological replicates; error bars indicate the standard deviation. Significance was determined by ANOVA; ^*^.01 < *P* < .05 and ^**^P < .01 were considered to indicate significant and highly significant levels, respectively.

RNAi of *SbMYB3* was carried out in *S. baicalensis* hairy roots to confirm its function. Since the RNAi vector carried a red fluorescence gene (*dsRED*), positive hairy root lines could be screened by observation of red fluorescence. We obtained several hairy root lines with red fluorescence and screened two hairy root lines with significantly downregulated *SbMYB3* expression levels. *SbMYB3* expression in the RNAi lines was 42.15% and 46.58% of that in the EV line, respectively. *SbMYB3* suppression decreased the levels of baicalin and wogonoside dramatically. In line 1 and line 10, baicalin levels were reduced to 1.75% and 32.90% of the control level, and wogonoside accumulation was reduced to 2.15% and 42.17% of that in the EV line, respectively. The content of baicalein was also reduced in the RNAi1 line, and was 43.90% of that in the EV line (Fig. 4A and Supplementary Data Fig. S8A). These data suggested that SbMYB3 is a positive regulator of RSF synthesis.

Clustered regularly interspaced short palindromic repeats (CRISPR)-Cas (CRISPR-associated protein)-mediated knockout of *SbMYB3* was also performed in *S. baicalensis* hairy roots to confirm the role of *SbMYB3*. To achieve gene editing of *SbMYB3*, we constructed two vectors: one vector carrying *dsRED* driven by the CaMV35S promoter, which served as the control group; and another vector carrying *dsRED* driven by the CaMV35S promoter, *Streptococcus pyogenes CAS9* driven by *A. thaliana* UBI10 promoter, and a guide RNA that targeted *SbMYB3* driven by the *A. thaliana* U6–26 promoter (Fig. 4B). We obtained one hairy root line with mutations in both alleles (Fig. 4C). The metabolic profiles indicated that *SbMYB3* knockout notably decreased the accumulations of baicalein, baicalin, wogonin, and wogonoside, which were 18.50%, 8.18%, 24%, and 16% of those in the EV line, respectively (Fig. 4D and Supplementary Data Fig. S8B). These expression profiles were in line with patterns of flavone accumulation, and the *SbMYB3* knockout downregulated the expression of most RSF biosynthetic genes, including *SbPAL2*, *SbPAL4*, *SbCLL7*, *SbCHS-2*, *SbFNSII-2*, *SbF6H*, *SbF8H*, *SbPFOMT5*, and *SbUBGAT*. Their transcript levels in the knockout line were 50%, 31.6%, 7.68%, 10.9%, 28.15%, 26.24%, 8.58%, 31.89%, and 50% of those in the EV line, respectively (Fig. 4E). These results suggested that *SbMYB3* knockout repressed expressions of most biosynthetic genes, thus decreasing RSF biosynthesis.

### *SbMYB3* overexpression enhanced RSF biosynthesis

**Figure 5:**
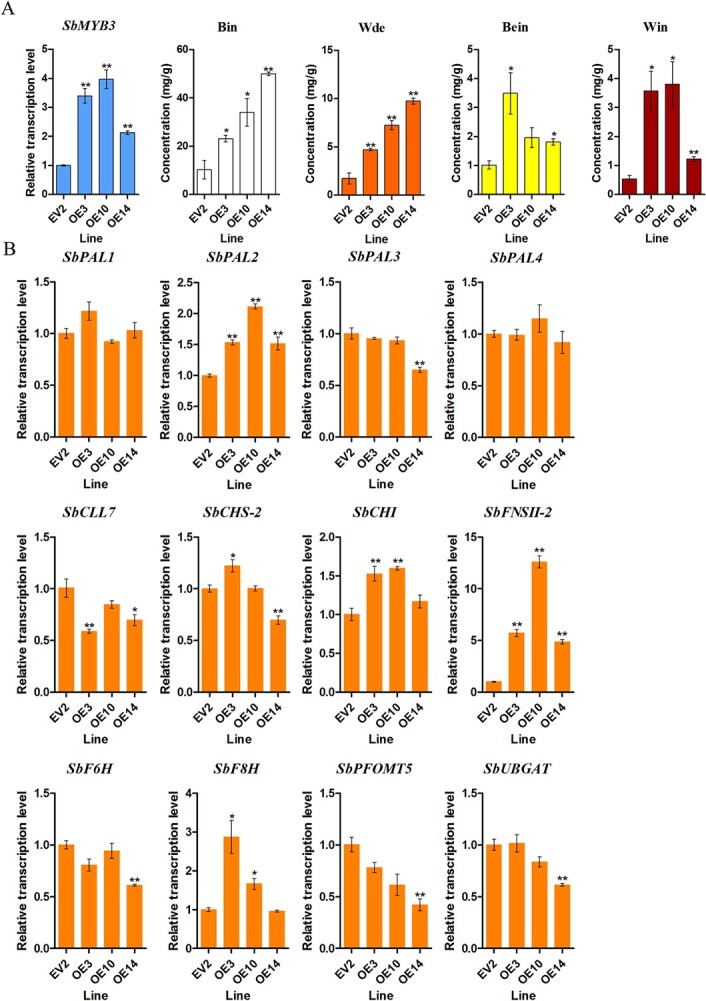
*SbMYB3* overexpression enhanced the biosynthesis of root-specific flavones in *S. baicalensis* hairy roots. (A) Expression analysis of *SbMYB3* and contents of baicalin, wogonoside, baicalein, and wogonin in *S. baicalensis* hairy roots. EV2 and OE represent hairy roots of the control line and *SbMYB3*-overexpression lines, respectively. Bin, Bein, Wde, and Win represent baicalin, baicalein, wogonoside, and wogonin, respectively. (B) Relative expression levels of key genes involved in root-specific flavone biosynthesis in *S. baicalensis* hairy roots. *SbPAL1*, *SbPAL2*, *SbPAL3* and *SbPAL4* encode phenylalanine ammonia lyases. *SbCLL7*, *SbCHS-2*, *SbCHI*, *SbFNSII-2*, *SbF6H*, *SbF8H*, *SbPFOMT5*, and *SbUBGAT* encode 4-coumaroyl:CoA-ligase, pinocembrin-chalcone synthase, chalcone isomerase, flavone synthase II-2, flavone 6- hydroxylase, flavone 8-hydroxylase, 8-*O*-methyl transferase, and flavonoid 7-*O*-glucosyltransferase, respectively. All data are the means of three biological replicates; error bars indicate the standard deviation. Significance was determined by ANOVA; ^*^.01 < *P* < .05 and ^**^P < .01 were considered to indicate significant and highly significant levels, respectively.

To further verify whether SbMYB3 functions as a positive regulator for RSF biosynthesis, *SbMYB3* overexpression lines (*35S*::SbMYB3) of *S. baicalensis* hairy roots were also generated. The expression profile indicated that *SbMYB3* expression was upregulated in the overexpression lines, and was 2.13- to 3.97-fold higher than that in the EV line. *SbMYB3* overexpression enhanced the contents of baicalein, baicalin, wogonin, and wogonoside, which were 1.77- to 3.42-, 2.25- to 4.87-, 2.32- to 7.19-, and 2.71- to 5.60-fold of those in the EV line, respectively (Fig. 5A and Supplementary Data Fig. S9). *SbFNSII-2* expression was consistent with alterations of flavone accumulation, and its level in the overexpression lines was 4.88- to 12.61-fold higher than that in the EV line, respectively. Moreover, *SbF8H* expression was also upregulated (Fig. 5B). These results suggested that SbMYB3 enhanced RSF biosynthesis by direct activation of *SbFNSII-2* expression.

## Discussion

In medicinal plants, MYB transcription factors are involved in the regulation of various specialized metabolites, such as tanshinones [33], phenolic acids [34], and artemisinin [35]. R2R3-MYBs comprise the largest MYB subfamily in plants and play vital roles in the regulation of specialized metabolism in plants, including regulation of the metabolic pathways of shikimate, terpenoid, flavonoid, and monolignol biosynthesis [11, 36, 37]. Based on the conserved amino acid sequence motifs, they are classified into 28 subgroups [17]. In *Arabidopsis*, R2R3-MYBs in subgroup 20 are involved in stress responses (AtMYB2, AtMYB62, and AtMYB112), gibberellic acid biosynthesis (AtMYB62), jasmonate-mediated stamen maturation (AtMYB108), and regulation of anthocyanin formation (AtMYB112) [26, 30, 38–40]. AtMYB62 has been reported to regulate phosphate starvation responses and gibberellic acid biosynthesis [26]. In this study, a new function (regulation of 4′-deoxyflavone biosynthesis) of SbMYB3, a close homolog of AtMYB62, was elucidated in *S. baicalensis*. We demonstrated that the SbMYB3 transcription factor can *trans*-activate the *SbFNSII-2* promoter, resulting in the considerable enhancement of RSF production in *S. baicalensis*. SbMYB3 is identical to SbMYB12 from a previous study, showing that its expression could be upregulated by treatments with abscisic acid, methyl jasmonate, and drought [41]. These results suggested that SbMYB3 is also involved in plant hormone signaling and stress responses.

Core biosynthetic genes and transcription factors involved in specialized metabolism are specifically expressed in different tissues of medicinal plants, where bioactive ingredients accumulate abundantly. RSFs are highly accumulated in *S. baicalensis* roots [5]. Based on the consistency of metabolic distribution and gene expression patterns, root-specific SbMYB transcription factors are candidates for regulating RSF biosynthesis (Fig. 1A). Therefore, we isolated the coding regions of six root-specific *SbMYBs* (Supplementary Data Tables S1–S3). *SbFNSII-2* is also root-specific and plays a crucial role in RSF biosynthesis [5]. The *SbFNSII-2* promoter was used to search for upstream transcription factors by yeast one-hybrid assays. We found that SbMYB3 shows strong interaction with the *SbFNSII-2* promoter (Fig. 2 and Supplementary Data Fig. S4). *SbFNSII-1.2* and *SbFNSII-2* are separately responsible for flavone biosynthesis in the aerial parts and roots [5], and their promoters both have MBS, MRE, and MYB motifs, but the *SbFNSII-1.2* promoter could not be bound by the SbMYB3 transcription factor (Supplementary Data Figs S3, S5, and S6). These results suggested that SbMYB3 may have evolved to regulate the RSF synthesis pathway. Interestingly, the *SbFNSII-2* promoter has a hAT transposon containing the MYB motif, but no Class II transposable element was found in the *SbFNSII-1.2* promoter (Supplementary Data Figs S3 and S5), suggesting that the hAT transposon may confer new root-specific regulation.

Plant R2R3-MYB transcription factors normally promote flavonoid accumulation by activating expressions of key genes in the flavonoid pathway. In *Arabidopsis*, AtMYB12 regulates flavonol synthesis mainly in the roots by activating the expressions of *CHS*, *CHI*, *FLAVANONE 3-HYDROXYLASE*, and *FLAVONOL SYNTHASE 1* (*FLS1*) [42]. AtMYB21 and its homologs AtMYB24 and AtMYB57 enhance flavonol accumulation through regulation of *FLS1* expression in the stamen [43]. Our work indicated that the SbMYB3 transcription factor enhanced the activity of the *SbFNSII-2* promoter, which was also confirmed by the expression pattern of *SbFNSII-2* under the control of SbMYB3 (Figs 3–5).

RNAi of *SbMYB3* led to substantial reductions in baicalin and wogonoside accumulation, suggesting that *SbMYB3* is a transcriptional activator of RSF biosynthesis (Fig. 4A). We confirmed this using CRISPR-CAS gene editing in *S. baicalensis* hairy roots to generate the *SbMYB3* knockout line. Analysis of one line with two knockout alleles confirmed the gene silencing results and showed that knocking out *SbMYB3* decreased the accumulation of baicalein, baicalin, wogonin, and wogonoside dramatically by downregulation of transcripts of *SbPAL2*, *SbPAL4*, *SbCLL7*, *SbCHS-2*, *SbFNSII-2*, *SbF6H*, *SbF8H*, *SbPFOMT5*, and *SbUBGAT*, confirming the role of SbMYB3 in regulating RSF biosynthesis (Fig. 4B–E). Although *SbMYB3* overexpression notably enhanced RSF biosynthesis, only the expressions of *SbFNSII-2* and *SbF8H* were upregulated, and *SbFNSII-2* expression was upregulated significantly (Fig. 5). These results suggested that SbMYB3 activates RSF biosynthesis by direct control of *SbFNSII-2* transcription. In *Gentiana triflora*, GtMYBP3 and GtMYBP4, which belong to P1/subgroup 7 (flavonol-specific MYBs), enhance the promoter activity of *GtFNSII* and regulate early flavonoid biosynthesis in gentian flowers [44]. CmMYB012 suppresses flavone biosynthesis in response to high temperatures in chrysanthemum by direct regulation of *CmFNS* [45]. AgMYB12 binds to the *AgFNS* promoter and activates *AgFNS* expression, thus promoting apigenin biosynthesis in celery [46]. These results suggested that *FNS* is an important target gene which might be regulated by R2R3-MYBs involved in flavonoid biosynthesis. The number of RSF biosynthetic genes regulated by *SbMYB3* overexpression was less than that regulated by its knockout (Figs 4E and 5B). The inconsistency may be due to the fact that the upregulation of *SbMYB3* expression level was much smaller than the downregulation caused by its knockout.

Taking these results together, gain or loss of function of SbMYB3 led to increased or decreased RSF accumulations, confirming that SbMYB3 is a positive regulator for RSF biosynthesis. The yeast one-hybrid assay and transcriptional activation assay demonstrated that the SbMYB3 transcription factor interacts with the *SbFNSII-2* promoter and enhances its activity. Expression profiles also indicated that SbMYB3 upregulates the expression of *SbFNSII-2*.

## Materials and methods

### Cloning and characterization of *SbMYBs* and the promoters of *SbFNSII-1.2* and *SbFNSII-2*

Based on the transcriptome data of different organs and the genome of *S. baicalensis* [5, 9], we amplified the full-length coding regions of *SbMYBs*, and isolated the promoters of *SbFNSII-1.2* and *SbFNSII-2* using specific primers (Supplementary Data Table S1). Gene locus IDs of the isolated *SbMYBs* are shown in Supplementary Data Table S2. These cDNAs and promoters were constructed into vector pDONR207 and were validated by complete sequencing (Tsingke, China).

### Yeast one-hybrid assay

The Matchmaker™ Gold yeast one-hybrid system (Clontech, Japan) was applied to screen the candidate transcription factors. The *SbFNSII-1.2* and *SbFNSII-2* promoters were separately constructed into vector pAbAi as two baits. Recombinant plasmids of *proSbFNSII-1.2-*pAbAi and *proSbFNSII-2-*pAbAi were separately digested with BbsI restriction endonuclease. Then, linearized plasmids of *proSbFNSII-1.2-*pAbAi and *proSbFNSII-2-*pAbAi were integrated into the genome of Y1HGold yeast, forming two bait-reporter yeast strains. The open reading frames of different *SbMYBs* were constructed into the vector pGADT7 as preys. Different recombinant plasmids of *SbMYBs*-pGADT7 were separately transformed into the bait strain, while the strain carrying an empty pGADT7 plasmid served as the negative control. A feasible inhibitory concentration of aureobasidin A (AbA) was applied in SD/−Leu medium to screen for transcription factors that bind to the bait. Yeast strains carrying different preys were cultured for 3 days at 28°C. Deletion analysis of the *SbFNSII-2* promoter was also performed using the above-mentioned methods.

### Transcriptional activation assay

Three vectors were designed to determine if SbMYB3 is a transcriptional activator. The first vector only expresses GFP driven by the UBI5 promoter and the second vector contains GFP driven by the UBI5 promoter and GUS driven by the native *SbFNSII-2* promoter, and the two vectors served as control groups. The third vector expresses the same DNA regions as the second vector plus SbMYB3 driven by the CaMV35S promoter. The three vectors were separately transformed into *A. rhizogenes* Ar1193. Then, successful transformants were used for induction of tobacco hairy roots. Finally, different lines of tobacco hairy roots were analyzed by GUS histochemical assays and real-time quantitative PCR (qRT–PCR).

### Protein subcellular localization

The full-length cDNA (removing termination codon) of *SbMYB3* was cloned into transient expression vector pBINPLUS.GFP4 and fused with the *GFP* gene. Plasmids of empty pBINPLUS.GFP4 and *SbMYB3*- pBINPLUS.GFP4 were introduced into *A. rhizogenes* GV3101. Positive *Agrobacterium* transformants were cultured in YEB medium until the OD_600_ value reached 1, and centrifuged to discard the supernatant. *Agrobacterium* sediments were resuspended with 10 mM MgCl_2_ and the OD_600_ was adjusted to 1. Then, the *Agrobacterium* suspensions were supplemented with acetosyringone to the concentration of 100 μM and stood at room temperature for 3 hours. *Agrobacterium* suspensions were applied to inject young leaves of tobacco. After the injection, the tobacco was cultured in the dark for 1 day, followed by light culture for 3 days. GFP signals were observed by confocal laser microscopy (Olympus FV10i) and nuclei were stained with DAPI (Leagene, China).

### Transgenic hairy root cultures

The full-length cDNA and a 300-bp fragment (non-conserved region) of *SbMYB3* were introduced into pK7WG2R and pK7GWIWG2R vectors by Gateway technology, respectively. A knockout vector of *SbMYB3* mediated by CRISPR was also constructed using Goldengate technology [47]. The overexpression and RNAi plasmids were transformed into *A. rhizogenes* A4. Due to conflict of resistance between the knockout vector and *A. rhizogenes* A4, *A. rhizogenes* MSU440 was used to introduce the knockout plasmid. Based on a previous protocol [5], the different positive *Agrobacterium* transformants were applied to infect *S. baicalensis* leaves to induce hairy roots. Positive lines of hairy roots were confirmed by red fluorescence inspection, as the positive overexpression, RNAi, and knockout lines expressed dsRED protein. Different lines and control lines of *S. baicalensis* hairy roots were cultured for 50 days and collected. The hairy roots were ground into powder; one part was freeze-dried to extract flavones and another part was used for RNA isolation.

### Flavone extraction and HPLC analysis

Lyophilized hairy root powder (1.5 mg) was ultrasonically extracted with 1.5 ml of 70% methanol for 2 hours, and the powder residue was removed by centrifugation. Flavone extracts were filtered with 0.22-μm filters and then analyzed using the Agilent 1260 Infinity II HPLC system. According to a previous method [5], a 100 × 2-mm 3 μm Luna C18 (2) column was used for separation. Flavones were detected at 280 nm. Based on the retention time of standard substances and standard curves, flavones were confirmed and measured.

### Relative expression analysis by qRT–PCR

According to the protocol of the RNAprep Pure Plant Kit (TIANGEN, China), 50 mg of hairy root powder was used to isolate total RNA. cDNA was synthesized from 500 ng of RNA using the PrimeScript™ RT Master Mix (Takara, Japan). qRT–PCR was performed using TB Green^®^ Premix Ex Taq™ II (Takara, Japan) and specific primers (Supplementary Data Table S1). The 2^−ΔΔCT^ method was used to calculate transcript levels of the relevant genes.

## Acknowledgements

This work was supported by the National Key R&D Program of China (2018YFC1706200), the National Natural Science Foundation of China (31870282 and 31700268), the Chenshan Special Fund for Shanghai Landscaping Administration Bureau Program (G182401, G192419, and G212401), and the Youth Innovation Promotion Association, Chinese Academy of Sciences. Q.Z. is also supported by a Sanofi-SIBS scholarship and C.M. is supported by the CAS/JIC Centre of Excellence for Plant and Microbial Sciences (CEPAMS) joint foundation. We thank Dr Ping Xu, Dr Lei Yang, and Dr Jingjing Xu from Chenshan Plant Science Research Center for their advice on the experiments.

## Author contributions

Q.Z. and Y.M.F. conceived and designed the study; Y.M.F. and C.M. performed bioinformatics analyses; Y.M.F., J.L., M.M.Z., S.M.Z., T.L.P., M.Y.C., L.J.C., and H.W.X. performed the experiments; Y.M.F., Q.Z., J.Y., and C.M. analyzed and interpreted the data; Y.M.F., Q.Z., and C.M. wrote the paper with significant input from all authors.

## Data availability

The sequences of the genes isolated in this study have been submitted to the Nucbank database (https://ngdc.cncb.ac.cn/nucbank/) with the accession numbers *SbMYB1*, C_AA001102.1; *SbMYB2*, C_AA001103.1; *SbMYB3*, C_AA001104.1; *SbMYB4*, C_AA001105.1; *SbMYB5*, C_AA001106.1; and *SbMYB6*, C_AA001107.1.

## Conflict of interest

The authors have declared no conflicts of interest.

## Supplementary data

Supplementary data is available at *Horticulture Research* online.

## Supplementary Material

Web_Material_uhac266
