# Supplementary material for: SbMYB3 transcription factor promotes root-specific flavone biosynthesis in Scutellaria baicalensis
Source: Hortic Res. 2022 Dec 2;10(2):uhac266. doi: 10.1093/hr/uhac266 (PMC9909510; doi:10.1093/hr/uhac266)
Supplement: Web_Material_uhac266 [file web_material_uhac266.docx]

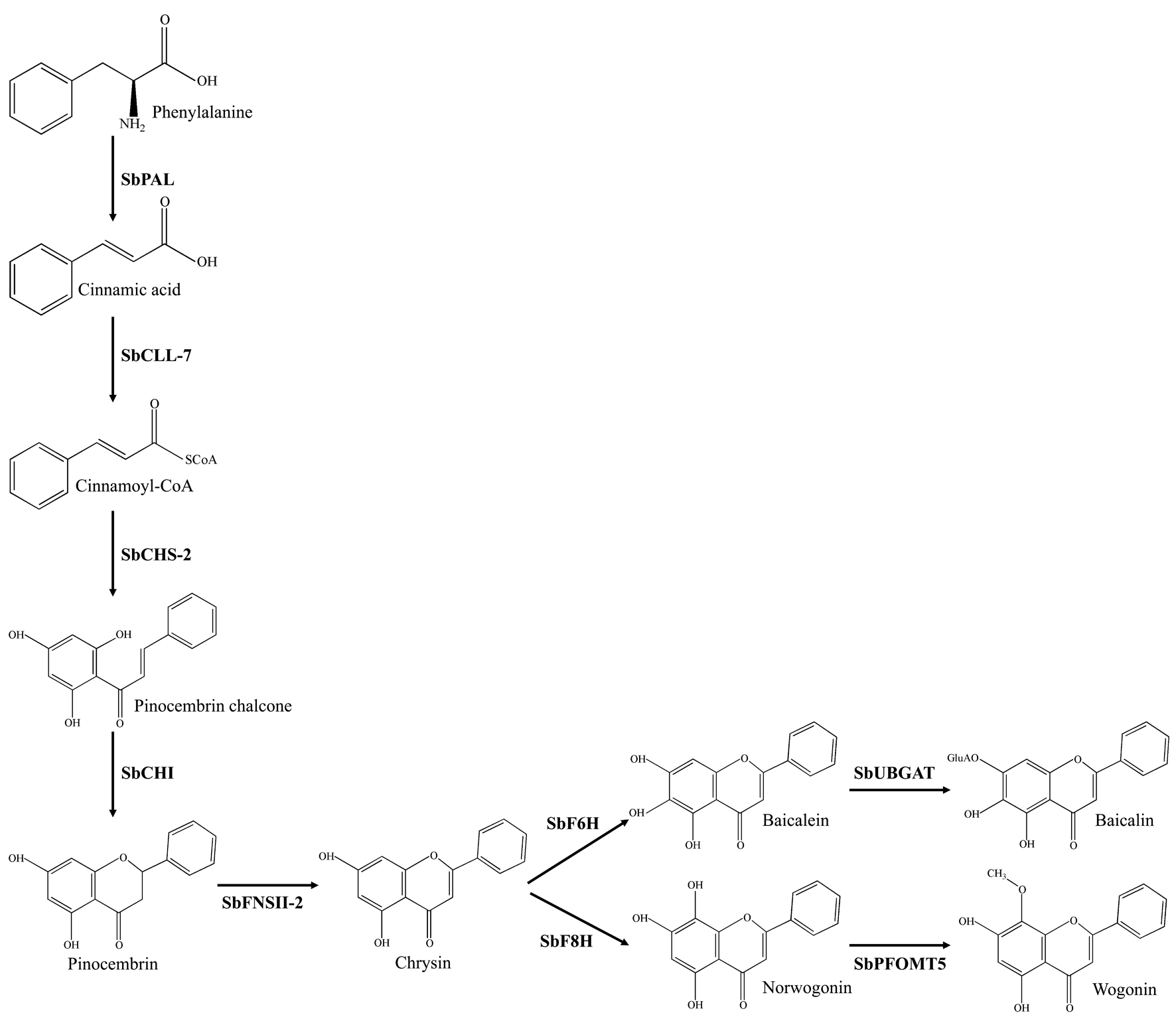


Figure S1. The biosynthetic pathway of root-specific flavone in *Scutellaria baicalensis*.

SbPAL, SbCLL7, SbCHS-2, SbCHI, SbFNSII-2, SbF6H, SbF8H, SbPFOMT5, and SbUBGAT encode phenylalanine ammonia lyase, 4-coumaroyl: CoA-ligase, pinocembrin-chalcone synthase, chalcone isomerase, flavone synthase II-2, flavone 6- hydroxylase, flavone 8-hydroxylase, 8-O-methyl transferase, and flavonoid 7-O-glucosyltransferase, respectively.


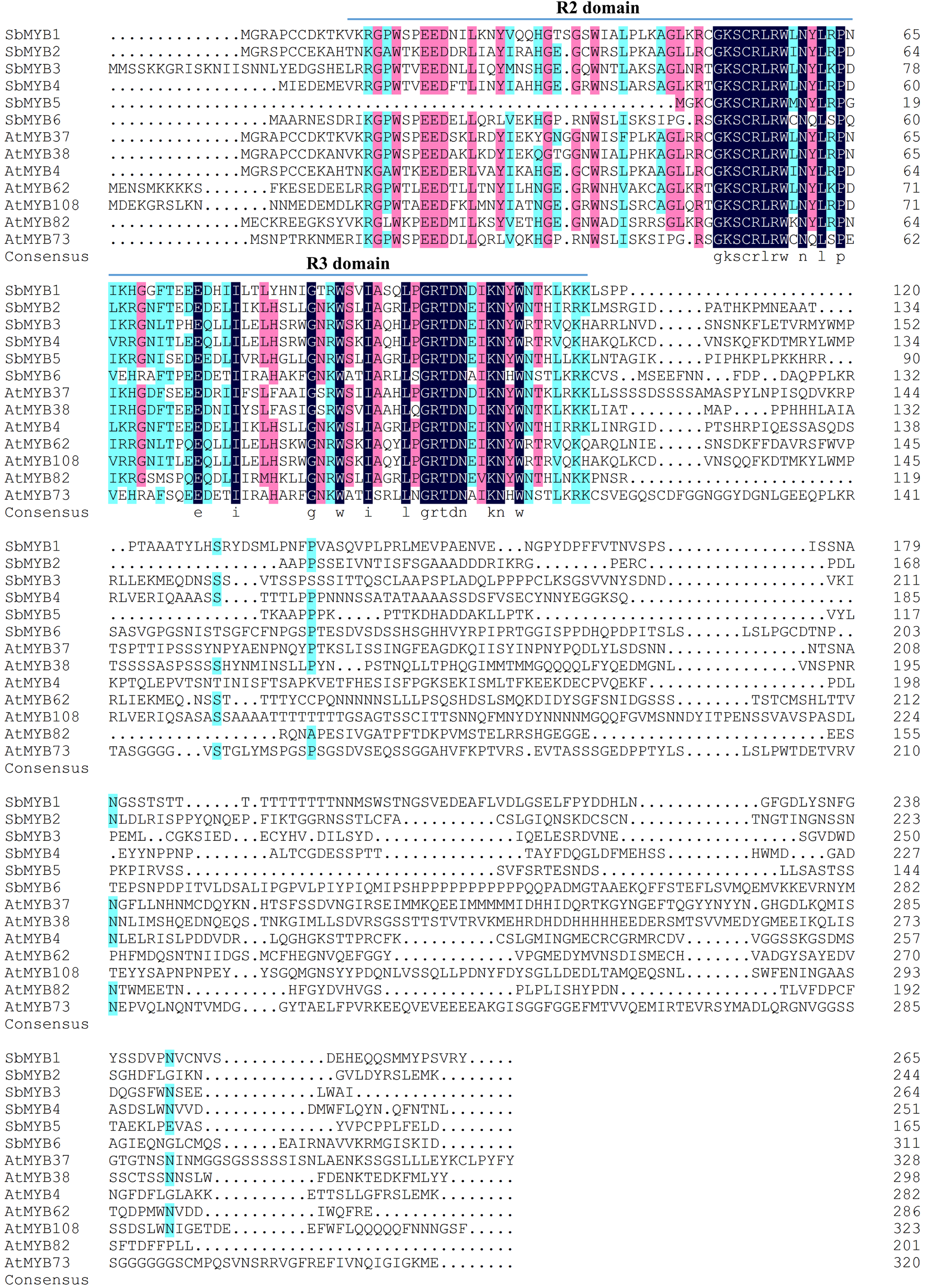


Figure S2. Full-length protein sequence alignment of SbMYBs and *Arabidopsis thaliana* MYBs.


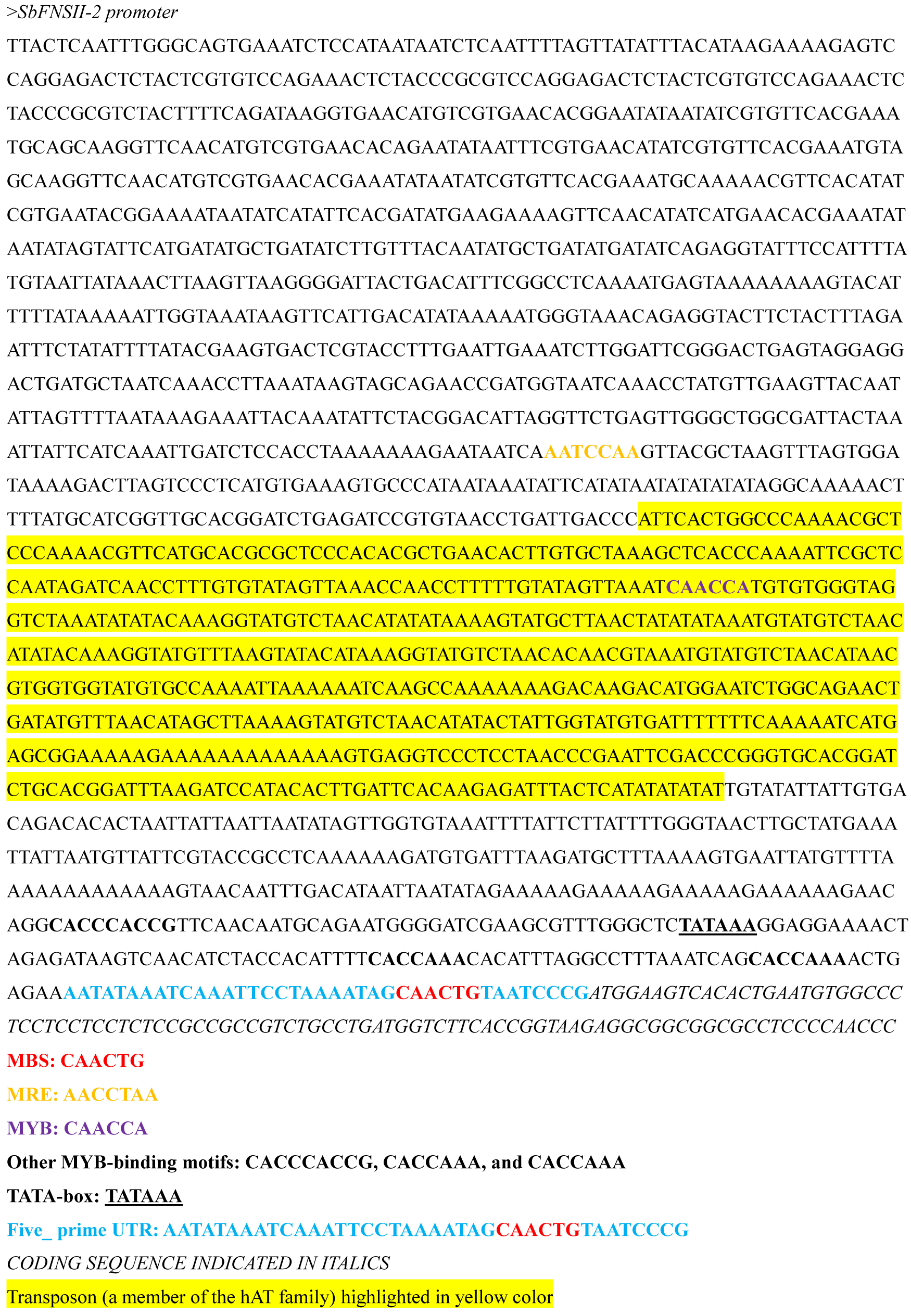


Figure S3. The motifs analysis of *SbFNSII-2* promoter using PlantCARE database and Plant Repeat Database. “MRE”, “MYB”, and “MBS” represent different MYB-binding motifs.


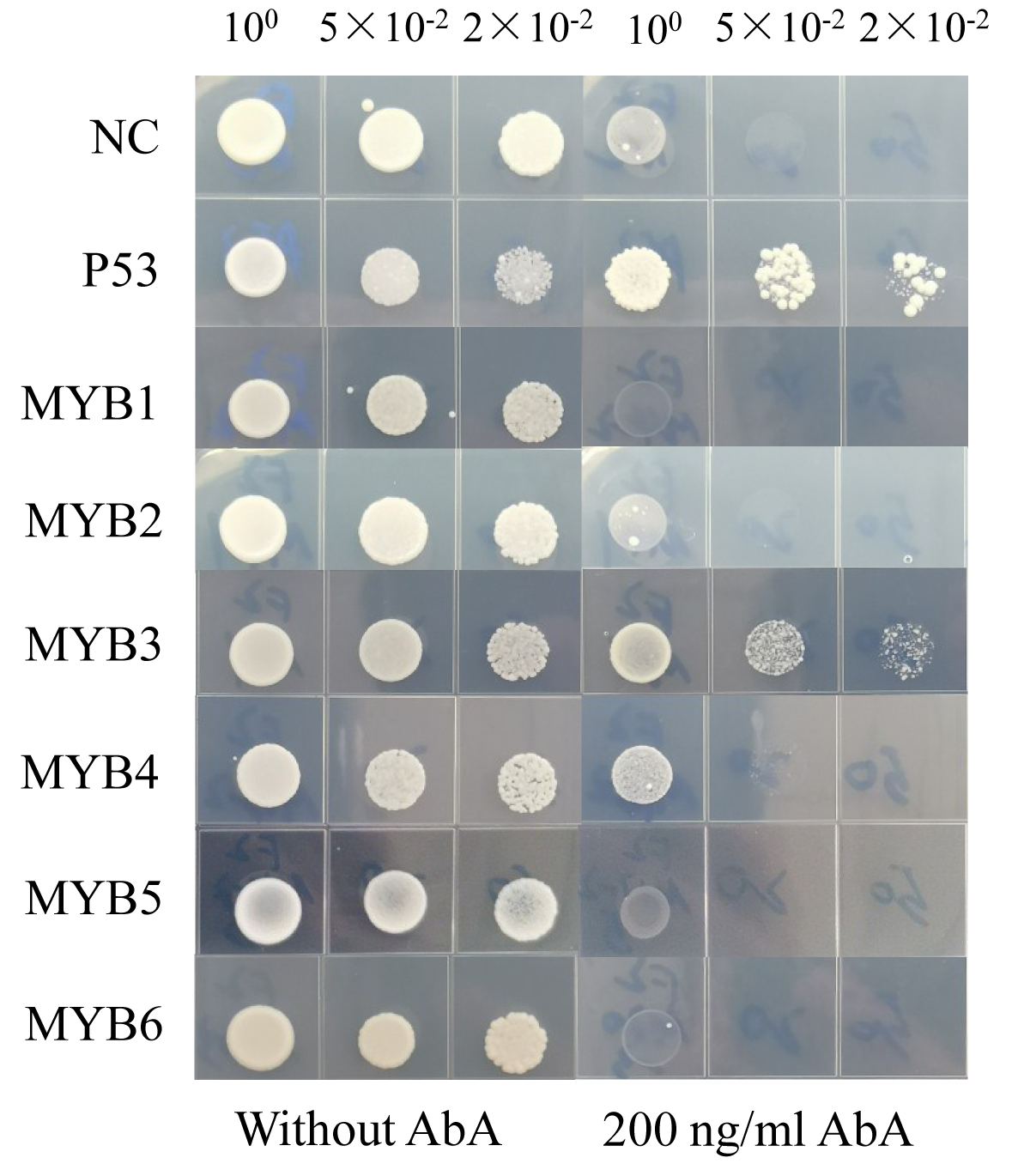


Figure S4. The interactions analysis between *SbFNSII-2* promoter and candidate SbMYBs using yeast one-hybrid assays. “NC” represents empty pGADT7 plus the *SbFNSII-2* promoter bait, whereas “P53” represents the positive control. “AbA” means Aureobasidin A.


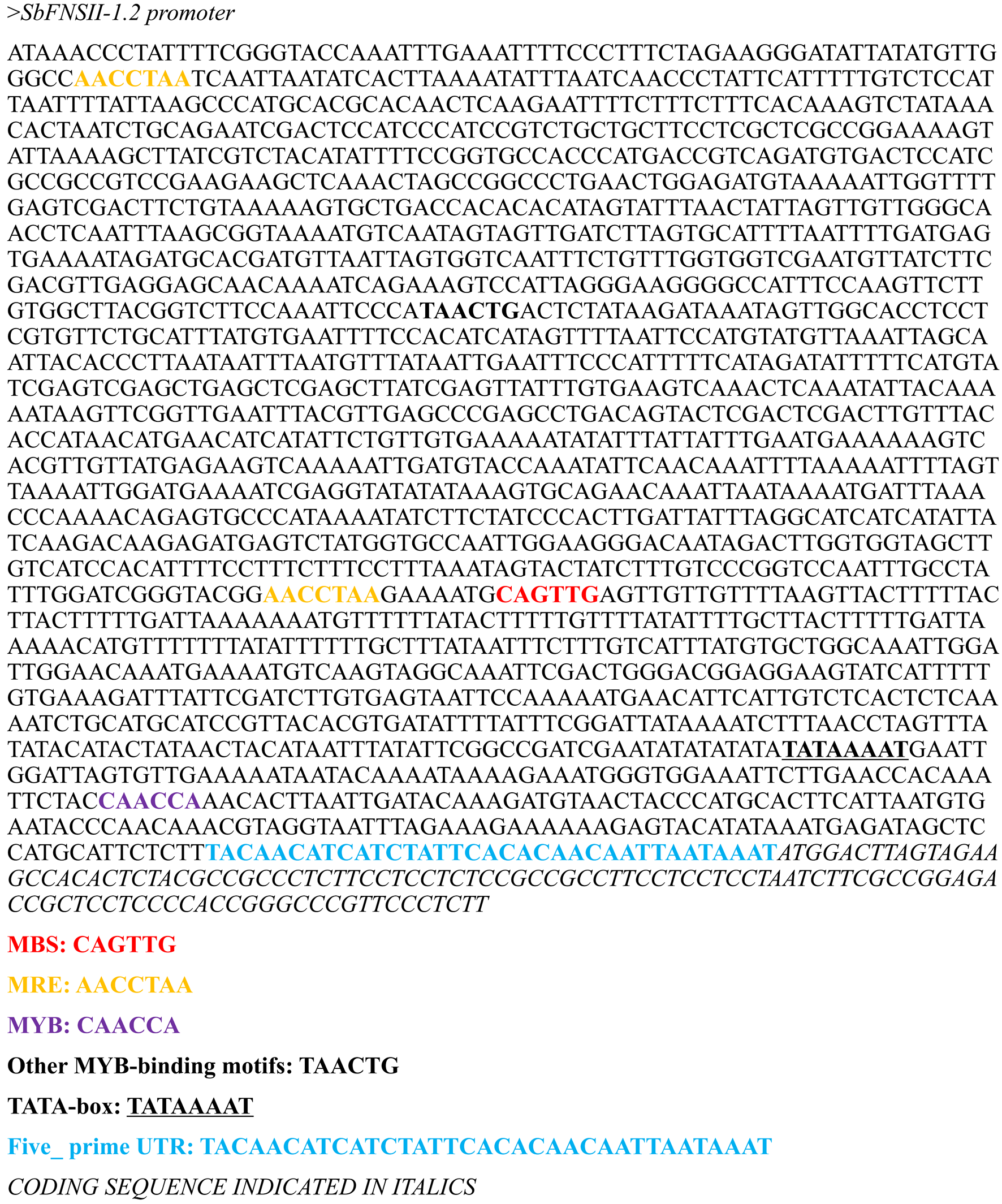


Figure S5. The motifs analysis of *SbFNSII-1.2* promoter using PlantCARE database. “MRE”, “MYB”, and “MBS” represent different MYB-binding motifs.


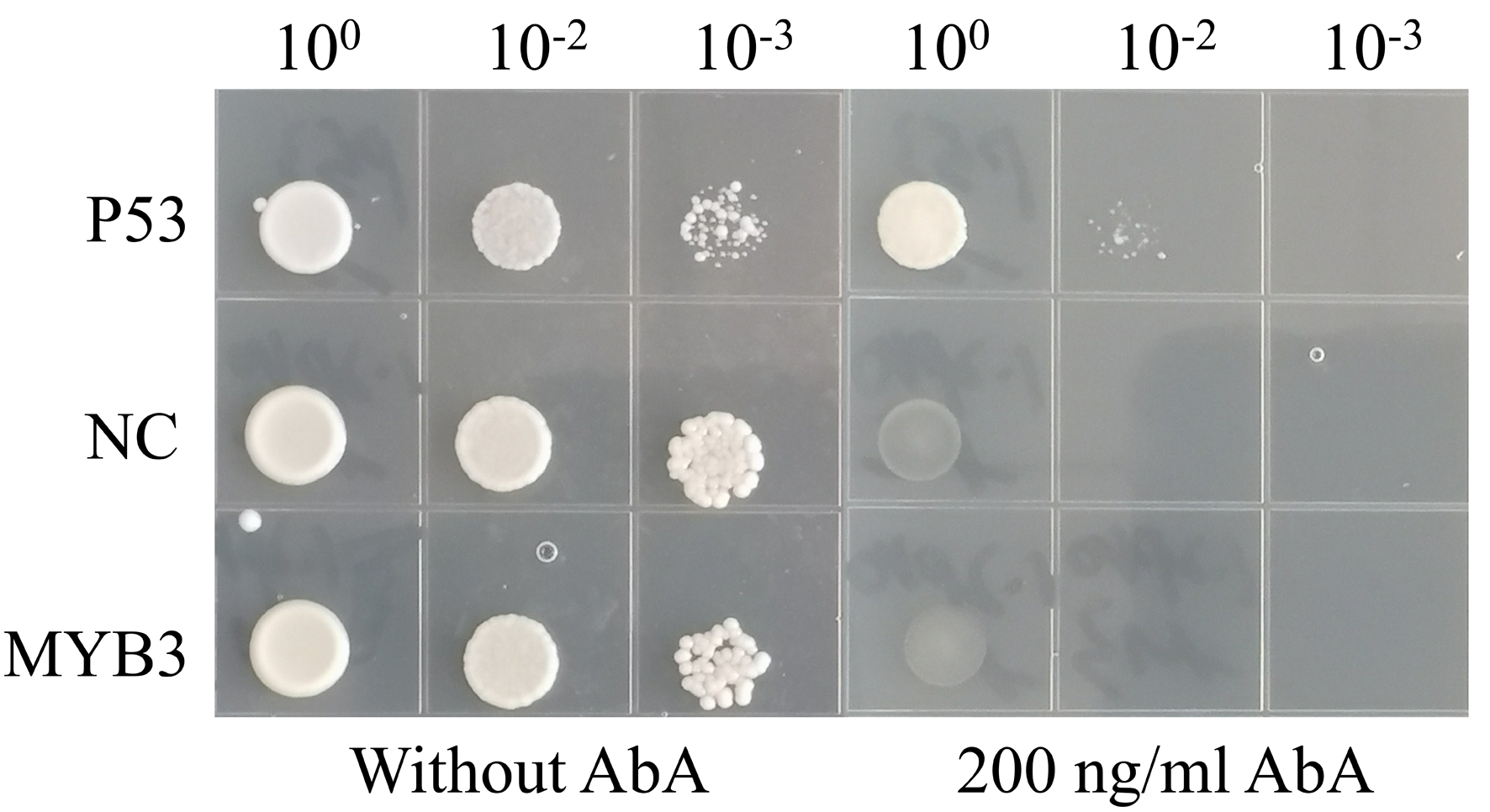


Figure S6. The interaction analysis between *SbFNSII-1.2* promoter and SbMYB3 transcription factor using yeast one-hybrid assays. “NC” represents empty pGADT7 plus the *SbFNSII-1.2* promoter bait, whereas “P53” represents the positive control. “AbA” means Aureobasidin A.


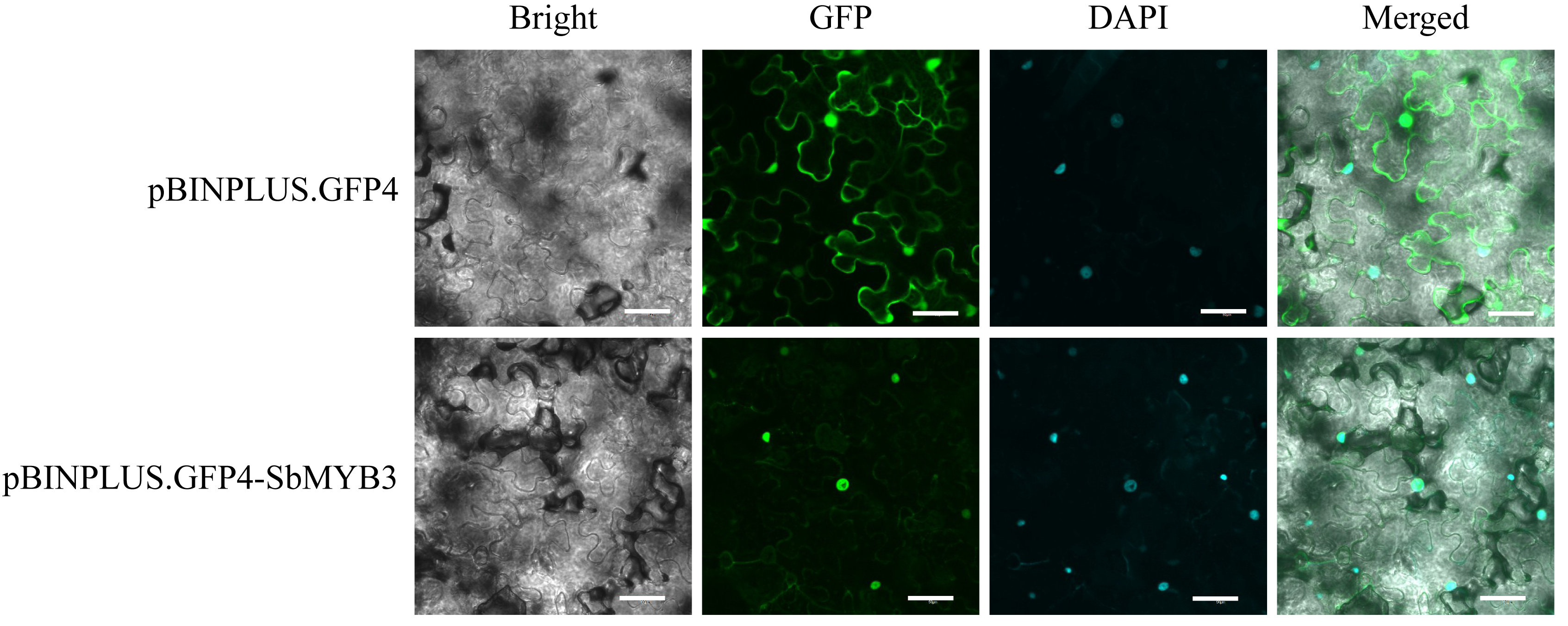


Figure S7. Subcellular localization of SbMYB3. The empty pBINPLUS.GFP4 and pBINPLUS.GFP4-SbMYB3 plamids were transiently expressed in tobacco leaves. Microscope inspection was performed at 4th day after inoculation. The pictures show bright field, green fluorescent field, DAPI field and merged results of three fields from left to right. Scale bars: 50 μm.


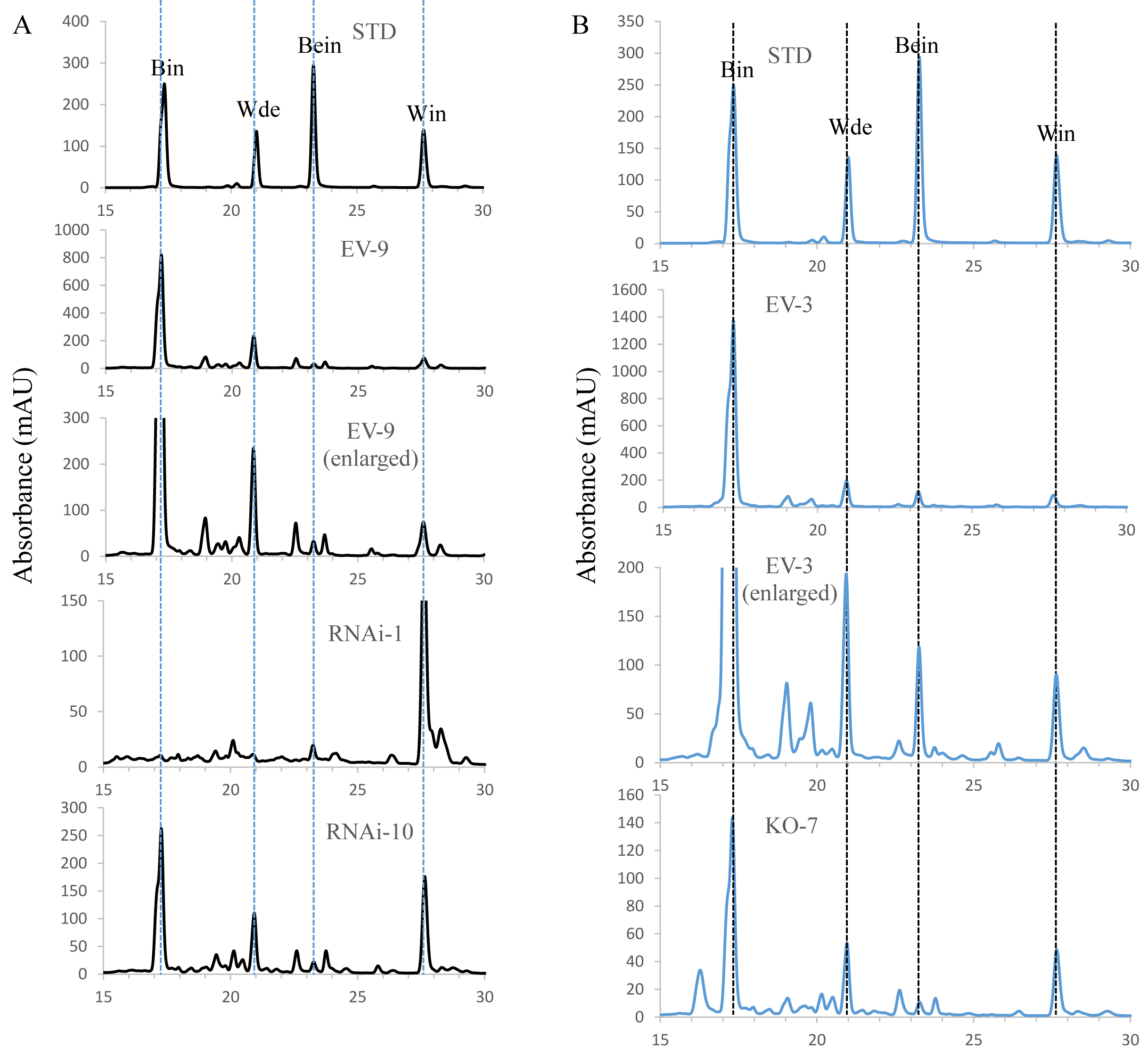


Figure S8. HPLC analysis of the RNAi lines, *SbMYB3* knockout line, and their control lines. (A) HPLC analysis of RNAi lines of *SbMYB3* and the control line. (B) HPLC analysis of *SbMYB3* knockout line and the control line. “STD”, “EV”, “RNAi”, and “KO” represent flavone standards, the control lines, RNAi lines of *SbMYB3*, and *SbMYB3* knockout line, respectively.“Bin”, “Bein”, “Wde”, and “Win” represent baicalin, baicalein, wogonoside, and wogonin, respectively.


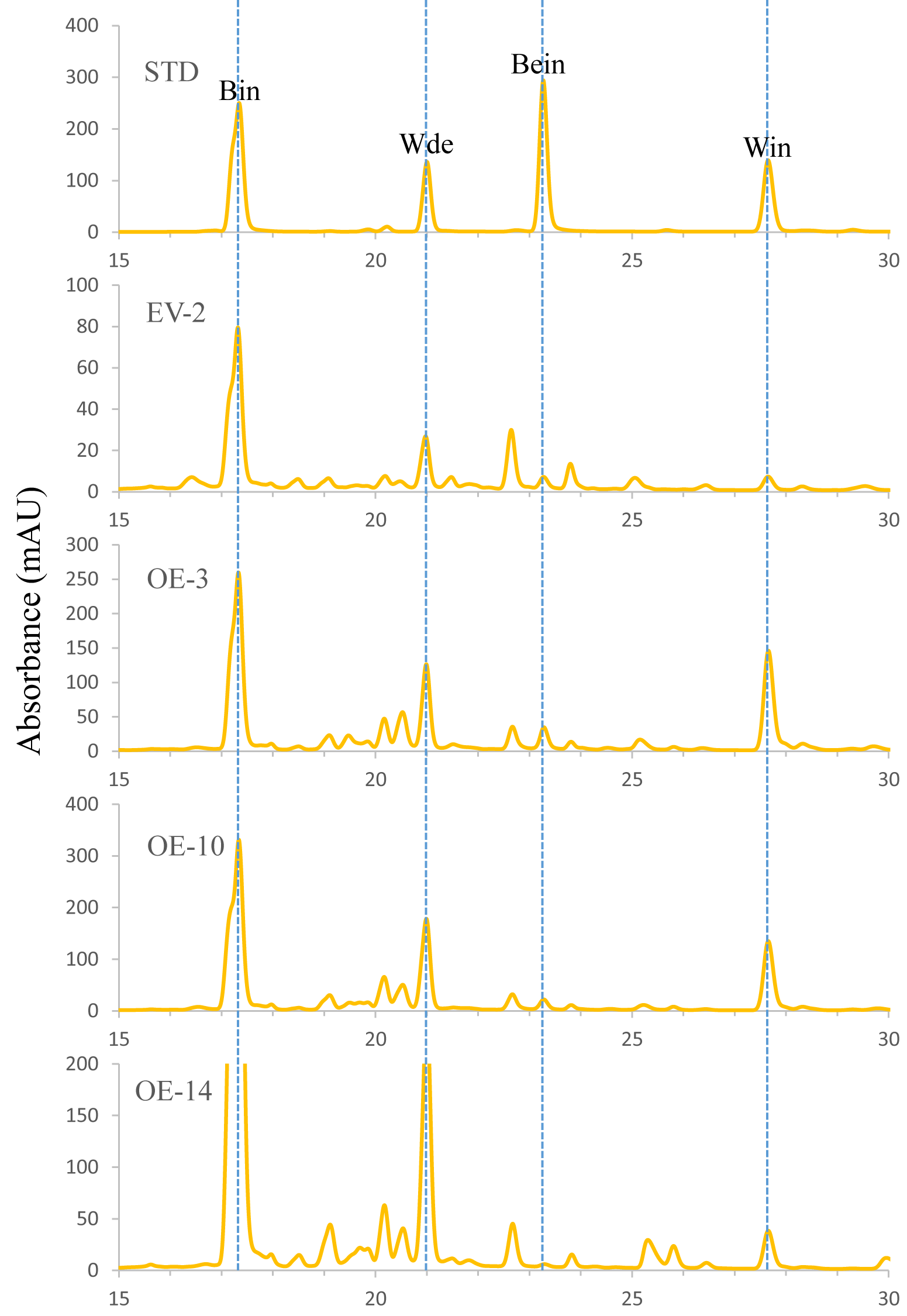


Figure S9. HPLC analysis of *SbMYB3* overexpression lines and the control line. “STD”, “EV”, and “OE” represent flavone standards, the control line, and *SbMYB3* overexpression lines, respectively. “Bin”, “Bein”, “Wde”, and “Win” represent baicalin, baicalein, wogonoside, and wogonin, respectively.

Table S1. The primers of genes used in this study

| **The name of primers** | **Sequence (5′–3′)** | **Application** |
| --- | --- | --- |
| *SbMYB1*-F | GGGGACAAGTTTGTACAAAAAAGCAGGCTTCATGGGAAGAGCACCTTGTTG | Cloning |
| *SbMYB1*-R | GGGGACCACTTTGTACAAGAAAGCTGGGTTTCAATATCTAACACTTGGATACATC | Cloning |
| *SbMYB2*-F | GGGGACAAGTTTGTACAAAAAAGCAGGCTTCATGGGGAGGTCTCCTTGTTGTG | Cloning |
| *SbMYB2*-R | GGGGACCACTTTGTACAAGAAAGCTGGGTTTCATTTCATCTCCAAGCTTCTG | Cloning |
| *SbMYB3*-F | GGGGACAAGTTTGTACAAAAAAGCAGGCTTCATGATGAGTAGTAAAAAAGGTAGAA | Cloning |
| *SbMYB3*-R | GGGGACCACTTTGTACAAGAAAGCTGGGTTCTAAATTGCCCATAATTCTTCAC | Cloning |
| *SbMYB4*-F | GGGGACAAGTTTGTACAAAAAAGCAGGCTTCATGATTGAAGATGAAATGGAGG | Cloning |
| *SbMYB4*-R | GGGGACCACTTTGTACAAGAAAGCTGGGTTCTAAAGGTTGGTGTTGAATTGG | Cloning |
| *SbMYB5*-F | GGGGACAAGTTTGTACAAAAAAGCAGGCTTCATGGGGAAGTGTGGAAAGAG | Cloning |
| *SbMYB5*-R | GGGGACCACTTTGTACAAGAAAGCTGGGTTTCAGTCGAGCTCAAAGAGC | Cloning |
| *SbMYB6*-F | GGGGACAAGTTTGTACAAAAAAGCAGGCTTCATGGCGGCGAGAAACGAATC | Cloning |
| *SbMYB6*-R | GGGGACCACTTTGTACAAGAAAGCTGGGTTTCAATCGATCTTGCTAATACCCATT | Cloning |
| *SbFNSII-2* promoter-F | GGGGACAAGTTTGTACAAAAAAGCAGGCTTCTTACTCAATTTGGGCAGTGAAATC | Cloning |
| *SbFNSII-2* promoter-R | GGGGACCACTTTGTACAAGAAAGCTGGGTTCGGGATTACAGTTGCTATTTTAG | Cloning |
| *SbFNSII-1.2 promoter-F* | GGGGACAAGTTTGTACAAAAAAGCAGGCTTCATAAACCCTATTTTCGGGTAC | Cloning |
| *SbFNSII-1.2 promoter*-R | GGGGACCACTTTGTACAAGAAAGCTGGGTTATTTATTAATTGTTGTGTGAATAG | Cloning |
| *SbFNSII-2* promoter-pAbAi-F | attcgagctcggtaccTTACTCAATTTGGGCAGTGAAATC | Yeast one-hybrid |
| *SbFNSII-2* promoter-pAbAi-R | cagatccccggcCGGGATTACAGTTGCTATTTTAG | Yeast one-hybrid |
| *SbFNSII-2* proP1-pAbAi-F | attcgagctcggtaccTTACTCAATTTGGGCAGTGAAA | Yeast one-hybrid |
| *SbFNSII-2* proP1-pAbAi-R | cagatccccggcTTTGATGAATAATTTAGTAATC | Yeast one-hybrid |
| *SbFNSII-2* proP2-pAbAi-F | attcgagctcggtaccTTGATCTCCACCTAAAAAAAGA | Yeast one-hybrid |
| *SbFNSII-2* proP2-pAbAi-R | cagatccccggcCGGGATTACAGTTGCTATTTTA | Yeast one-hybrid |
| *SbFNSII-2* proP3-pAbAi-F | attcgagctcggtaccACCTTTTTGTATAGTTAAATCA | Yeast one-hybrid |
| *SbFNSII-2* proP3-pAbAi-R | cagatccccggcCGGGATTACAGTTGCTATTTTA | Yeast one-hybrid |
| *SbFNSII-2* proP4-pAbAi-F | attcgagctcggtaccAAAAAGAAAAAGAAAAAAGAAC | Yeast one-hybrid |
| *SbFNSII-2* proP4-pAbAi-R | cagatccccggcCGGGATTACAGTTGCTATTTTA | Yeast one-hybrid |
| *SbMYB3*-GFP4-F | cgaacgatagggtaccATGATGAGTAGTAAAAAAGGTA | Subcellular localization |
| *SbMYB3*-GFP4-R | cccttgctcaccatggatccAATTGCCCATAATTCTTCACTG | Subcellular localization |
| *SbMYB3*RNAi-F | GGGGACAAGTTTGTACAAAAAAGCAGGCTTCGTGACGTCATCTCCATCTTCCTC | RNAi |
| *SbMYB3*RNAi-R | GGGGACCACTTTGTACAAGAAAGCTGGGTTCTAAATTGCCCATAATTCTTCACTG | RNAi |
| *SbPAL1*-QF | GCGAATAGTGTTCATGATGAGGAT | QPCR |
| *SbPAL1*-QR | CAATGGCTGCCTTTCCAGTT | QPCR |
| *SbPAL2*-QF | CAACAATGGGTTGCCTTCCA | QPCR |
| *SbPAL2*-QR | AAGCCATCGCGATTTCAGAC | QPCR |
| *SbPAL3*-QF | CTGAAATCGCCATGGCTTCA | QPCR |
| *SbPAL3*-QR | TCTGGACATGGTTCGTCACA | QPCR |
| *SbPAL4*-QF | ACTCGTTTAGCCGTTGCTTC | QPCR |
| *SbPAL4*-QR | CTTCGGAAGCCTTGAATCCG | QPCR |
| *SbCLL7*-QF | ATTGAGGCCACCGTTGTATC | QPCR |
| *SbCLL7*-QR | CGACTTTTGAAACCGTGGAT | QPCR |
| *SbCHS2*-QF | GCAGTCCACTTATGCTGATTAC | QPCR |
| *SbCHS2*-QR | GTGAAGTTGTCGTTCTCCTTC | QPCR |
| *SbCHI*-QF | AAGGCAGTAATAGAGAACAAACAG | QPCR |
| *SbCHI*-QR | TTAAAACAACTCCGATAGTCTTG | QPCR |
| *SbFNSII-2*-QF | TCACCTATGGCGTCTCCTTC | QPCR |
| *SbFNSII-2*-QR | CAGCTCCTCAGTGACGTTGA | QPCR |
| *SbF6H*-QF | CTACGACGGAGCCTCGCTTG | QPCR |
| *SbF6H*-QR | TGGCTCGCTGCAGCTCGATC | QPCR |
| *SbF8H*-QF | ATTGGACGGAATTGTTGGA | QPCR |
| *SbF8H*-QR | TCACTTCCACCCACAATCAA | QPCR |
| *SbPFOMT5*-QF | TTTGATGAATGTGCCTGCTGATGAGG | QPCR |
| *SbPFOMT5*-QR | CATGTTGGCCTTCTGAATGCTGG | QPCR |
| *SbUBGAT*-QF | CAACGGCCTATGCATTTCCA | QPCR |
| *SbUBGAT*-QR | TGTCGACATCTCCGACCAAT | QPCR |
| *SbMYB3*-QF | TGGAGAACAAGGGTGCAGAA | QPCR |
| *SbMYB3*-QR | AACCTGGGCATCCAGTACAT | QPCR |
| *GUS*-QF | ACATGGCATCGTGGTGATTG | QPCR |
| *GUS*-QR | CGCTTCGAAACCAATGCCTA | QPCR |
| *SbActin*-QF | TCTTGATCTTGCTGGTCGTG | QPCR |
| *SbActin*-QR | CACTGCAGAGCTGGTCTTTG | QPCR |
| *SbMYB3*-KO-TF | TGCAGGTTCAAATTCCATCTCTC | Gene editing tests |
| *SbMYB3*-KO-TR | TGGTAACATTCATCTTCAATCGATT | Gene editing tests |

The underlined sequences indicate recombination sites.

Table S2. Gene locus ID of the *SbMYBs* isolated by this study

| **Gene names** | **Gene locus ID** |
| --- | --- |
| *SbMYB1* | *Sb07g01790* |
| *SbMYB2* | *Sb02g34230* |
| *SbMYB3* | *Sb02g00440* |
| *SbMYB4* | *Sb08g00860* |
| *SbMYB5* | *Sb06g12420* |
| *SbMYB6* | *Sb08g11040* |

Table S3. The lengths of cDNAs and encoding proteins of isolated *SbMYBs* in this study

| **Gene names** | **cDNA lengths (bp)** | **Protein lengths (aa)** |
| --- | --- | --- |
| *SbMYB1* | 798 | 265 |
| *SbMYB2* | 735 | 244 |
| *SbMYB3* | 795 | 264 |
| *SbMYB4* | 756 | 251 |
| *SbMYB5* | 498 | 165 |
| *SbMYB6* | 936 | 311 |
